# Diagnosing migraine in research and clinical settings: The validation of the Structured Migraine Interview (SMI)

**DOI:** 10.1186/1471-2377-10-7

**Published:** 2010-01-14

**Authors:** Zainab Samaan, E Anne MacGregor, Dowson Andrew, Peter McGuffin, Anne Farmer

**Affiliations:** 1 Dept. of Psychiatry and Behavioural Neurosciences, McMaster University, Hamilton, Ontario, Canada; 2 The City of London Migraine Clinic and Research Centre for Neuroscience, Barts and the London School of Medicine and Dentistry, University of London, London, UK; 3 King's Headache Service, King's College Hospital, London, UK; 4 Medical Research Council Social Genetic and Developmental Psychiatry Centre, Institute of Psychiatry, King's College London, London, UK

## Abstract

**Background:**

Migraine is a common disorder that is highly co-morbid with psychopathological conditions such as depression and anxiety. Despite the extensive research and availability of treatment, migraine remains under-recognised and undertreated. The aim of this study was to design a short and practical screening tool to identify migraine for clinical and research purposes.

**Methods:**

The structured migraine interview (SMI) based on the International Classification of Headache Disorders (ICHD) criteria was used in a clinical setting of headache sufferers and compared to clinical diagnosis by headache specialist. In addition to the validating characteristics of the interview different methods of administration were also tested.

**Results:**

The SMI has high sensitivity (0.87) and modest specificity (0.58) when compared to headache specialist's clinical diagnosis.

**Conclusions:**

Our study demonstrated that a structured interview based on the ICHD criteria is a useful and valid tool to identify migraine in research settings and to a limited extent in clinical settings, and could be used in studies on large samples where clinical interviews are less practical.

## Background

It has been estimated that migraine is the second most prevalent brain disorder after anxiety, affecting nearly 41 million adult Europeans [1]. In a general population sample, 60% of migraine sufferers were not aware that they had migraine [2]. Only a small fraction of migraine sufferers were diagnosed. In one study, a diagnosis of migraine was only made for 41% of women and 29% of men who suffer with migraine headaches [3].

An even smaller fraction received treatment [4] with a very low percentage using prophylactic migraine therapy [5]. Even if they do seek help, almost 80% of patients with active migraine have no medical follow up [2]. Despite having effective treatment to relieve symptoms of acute migraine attacks such as triptans, the response rate to triptans varied from 44-70% and only Sumatriptan s.c. administration achieved 80% response rate [6]. In addition, triptans are expensive and not all patients have access to them. In a large population study of US households, only 20% of migraine sufferers used prescription treatment for acute migraine [7]. Migraine attacks were reported to occur at a rate of more than 3 per month in 31.3% of migraine sufferers and over half of migraine sufferers reported severe impairment. Over a quarter met the criteria for preventative treatment, however only 13% reported current use of such treatment [8].

Like psychiatric disorders, there are no known biological markers to identify migraine and the diagnosis is largely clinical, based on careful history taking and the absence of significant signs on physical examination. To avoid inconsistency and lack of agreement between clinicians and researchers, a structured and reliable method to define clinical syndromes and phenotypes such as depression and migraine, is needed to improve diagnostic accuracy [9].

In order to identify migraine in a systematic fashion, the International Headache Society (IHS) produced diagnostic criteria and classification system for primary headache disorders including migraine [10]. This provided a method for targeting a group of headache patients in need of care [11] to ensure accurate identification and a measurable way to assess outcome.

The majority of migraine epidemiological studies based on these criteria have provided a degree of harmony in identifying migraine. Nevertheless, the methodologies of case identification diverge greatly. Most epidemiological studies were conducted in the western world and showed migraine lifetime prevalence ranging from 10 - 28% [12][13][14][15][16][17][18]. The prevalence of migraine was much lower for a Japanese population (6%) [19]. More recent review of migraine prevalence studies worldwide (Africa, Asia, Australia, Europe, North America, Central and South America) that have been published since the introduction of the second edition of the International Classification of Headache Disorders (ICHD) [10] showed an average rate of 11% [20]. Although neurological and psychiatric disorders comprise only 1.4% of all deaths, they account for a remarkable 28% of all years of life lived with a disability [21]. The World Health Organization (WHO) estimated that there are 3000 migraine attacks occurring daily for every million population [22]. The WHO considered the disability from living with a day of a migraine attack to be similar to living with a day of quadriplegia [21].

Recognition of migraine is therefore important to reduce the symptoms' impact on patients as well as improving prognosis and associated psychiatric comorbidity. Migraine has been shown consistently to be associated with psychopathology such as depression [23] and anxiety [24] however; migraine is seldom screened for in psychiatric populations and barriers continue to exist that prevent the identification of migraine in different patients particularly in psychiatric populations.

The aim of the current study was to design a short and practical screening tool to identify migraine in settings where clinical interviews are not possible or practical such as large population studies and clinical practice where neurological expertise is limited.

In this study we report the validation of The Structured Migraine Interview (SMI) based on the ICHD criteria [10]. The purpose of the interview was to establish a lifetime ever diagnosis of migraine.

## Methods

The migraine interview was designed to answer the question of "did this person suffer from migraine at any time in his/her life?" Thus, questions were formulated to cover the individual items in the ICHD criteria, in the form of a brief 10 question structured interview. In addition to administration by an interviewer the questions could also be answered by self-report. As well as the presence of headache, enquiry was made about severity, frequency, duration, location, character, aura and other accompanying symptoms. The study was approved by the local ethics committee. The interview was then pilot tested on a small group of university staff members (n = 20, 70% were female, mean age 37.9 years, range 26-52). Participants were asked to comment on the relevance of the questions and any difficulties they had in completing the questionnaire. This information was then used to modify the interview into its final version (Additional file 1).

The average time required to complete the questions (by self report) was 5 minutes (range 3-10 minutes). The SMI consisted of 10 questions to yield the diagnosis of migraine with aura or migraine without aura. In addition the SMI included questions to distinguish probable migraine and non-migraine recurrent headaches.

The criteria for diagnosing migraine (ICHD) were as follows:

1. Migraine with aura (MA). At least 2 episodes of aura symptoms including visual disturbances prior to the onset of headache and fulfilling criteria for migraine, as in migraine without aura below.

2. Migraine without aura (MO). At least 5 episodes of moderate to severe headache with all of the following:

a) Pulsating or one-sided headache

b) Associated with nausea and/or vomiting and/or hypersensitivity to light or sound

c) Headache exacerbated by movement or similar physical activity

d) Headache lasting 4-72 hours

3. Probable Migraine (MP). At least 5 episodes of moderate to severe headache with only 3 of the features listed above under 2a-d.

4. Other headaches (OH). Recurrent headaches that do not fulfil the criteria for any form of migraine.

5. No recurrent headaches (NH).

The responses from the SMI were then scored using a computerized coding algorithm to generate the migraine diagnoses. The diagnosis of migraine with aura here is based on having the diagnostic features of migraine without aura that must include at least 5 attacks in a lifetime. In addition for the diagnosis of migraine with aura, there must be at least 2 attacks accompanied by aura symptoms as in question 4 (Additional file 1). In Additional file 1, question 3 we asked about hypersensitivity to sound or light while the ICHD criteria was worded to say "moderate to severe headache accompanied by hypersensitivity to sound AND light" This question is unlikely to over-diagnose migraine since a quarter of patients with migraine tend to under report these symptoms on routine questioning [25]. In addition each symptom alone is similarly correlated with migraine headache [26]. On the other hand, sensitivity to sound and light assessed alone was also found to be different predictors of migraine when compared to the gold standard (clinical diagnosis). This led to selecting only one (photosensitivity) for use in screening questionnaires [27]. It was also reported that the use of self-administered headache symptoms in a diary led to under-reporting of accompanying symptoms such as photophobia and phonophobia [28]. The use of this question 3 is therefore unlikely to have led to diagnosing MO in persons not fulfilling the criteria for MO. In question 7 (Additional file 1) we added "certain foods" as a result of the initial pilot were it was commonly reported that certain foods such as chocolate have triggered headache. This item was not considered as part of the diagnostic criteria. We also added question 10 that is not part of the ICHD criteria for 2 reasons. 1^st ^to identify any causes for headache to distinguish primary from secondary headaches and 2^nd ^to identify the proportion of people who were identified as migraine sufferers and if they actually knew that they had migraine.

## Recruitment of study subjects from the City of London Migraine Clinic for the validity and reliability studies

The City of London Migraine Clinic is a research centre and outpatient service for people with migraine and other headaches with 30 years of experience in treating headache disorders. Two of medical staff working in the clinic agreed to participate in the validity and reliability study of the SMI. The clinic receives referrals mainly from general practice but also from tertiary care throughout the UK. Patients attending the clinic were referred to the clinic because they suffered from significant headaches that were not managed by general practice and other health care providers. These patients were not all migraine sufferers but had other types of headaches. All patients who were registered with the clinic at the time of the study conduction (2003) were considered as potential study participants. In order to maintain confidentiality, information sheets and consent forms about the study, as well and the SMI questionnaires were sent directly by the clinic to their patients. Subjects who agreed to participate signed a consent form, and completed the SMI, returning both directly to the researcher (ZS) using the stamped addressed envelopes provided.

The clinic mailed out information about the study (December 2003) to their patients along with consent forms asking interested patients to take part in the study. Subjects who were willing to participate provided written informed consent and completed the SMI as well as demographic details.

The migraine diagnosis based on the SMI was compared with the migraine diagnosis assigned to the subjects by headache specialists. Migraine remains a clinical diagnosis and the clinical diagnosis is considered the "gold standard". Therefore in order to test the validity of the SMI compared to the diagnosis obtained from a headache specialist, a sub sample was selected. Two hundred subjects were randomly selected from migraine clinic attendees' respondents. The list of 200 subjects was then sent to the Migraine Clinic headache specialist to provide the clinical diagnoses. For practical reasons (time and resources available at the time of the study conduct) we selected a random sample form the original sample to test for the validity of the questionnaire. The selection ensured an equal opportunity for each participant to be selected by generating a random list of 200 study ID numbers that were assigned to participants arbitrary to conceal subjects' identity. This random and blind selection ensured a representative sample to be selected from the original study participants.

The study also tested the use of the SMI comparing face to face interview with an interviewer administered telephone interview. Twenty randomly selected subjects from the Depression Case Control migraine study [23] who had been interviewed face to face to complete the SMI were re-interviewed by telephone to obtain a life time diagnosis of migraine. The time between the face to face and telephone interviews was a mean of 2.5 years (range 1-4). Fifty subjects were randomly selected by the senior author blinded to migraine status in order to generate at least 20 subjects for comparison.

The data were entered into SPSS statistical package version 16 [29]. Statistical analysis was performed to estimate sensitivity and specificity of the SMI. Bivariate correlations were used to estimate the strength and direction of associations between SMI diagnoses, and the use of migraine pharmacological treatment and self reported migraine diagnoses.

## Results

Six hundred and forty six subjects completed the study data forms between December 2003 and April 2004. Demographic details are described in Table 1. Women represented the majority of subjects (82%) and the mean age of the study subjects was 48.6 years (SD 11.6).

**Table 1:** Demographic data of study participants (n = 646)

Ethnicity	Caucasian	Indian	Black			
	96%	2%	2%			

**Education**	Post Graduate	University	National diploma/Certificate	O or A Level	CSE	No qualification

	5.7%	9.8%	2.7%	55.6%	17.5%	8.8%

**Occupation**	Professional	Technical/Secretarial	Full time Student	Home making	Self employed	Unemployed

	51.2%	21.4	1.7%	11.9%	7.1%	6.7%

Seventy three percent of participants reported having migraine and 60% were receiving migraine pharmacological treatment. Just over half of participants were also taking over the counter analgesics. According to the SMI, 54.2% of subjects were diagnosed with migraine (with or without aura) in this sample.

Nonparametric correlations were used (the Test of Normality showed that the data distribution violated the assumption of normality p = 0.012). The diagnosis obtained by the SMI was significantly correlated with self-reported migraine (Spearman's rho = 0.16, p = 0.001). The SMI diagnosis was also significantly associated with taking migraine treatment (Spearman's rho = 0.35, p < 0.0001) and analgesics use (Spearman's rho = 0.12, p = 0.003).

Two hundred subjects were randomly selected (by senior author AF) from the migraine probands data base maintaining blindness to their migraine diagnoses. These subjects were then sent to the Migraine Clinic headache specialist to provide the clinical diagnoses. The headache specialist was able to verify 170 (85%) subjects' diagnoses. The clinical diagnosis included only 3 categories: migraine with aura 30.3%, migraine without aura 65.2% and other non-migraine headache 4.5%. In addition many cases of migraine with aura also had migraine without aura. There were no cases of probable migraine according to the clinical diagnosis. Therefore we combined the clinical diagnostic categories into migraine to include migraine with and without aura and non-migraine headaches. We designed the questionnaire with different types of migraine (with aura, without aura and probable migraine) in mind and we have included the ICHD criteria pertaining to the different types at the 2-digit level. However based on the clinical diagnosis, there were no cases of probable migraine diagnosed and most cases of migraine with aura were also diagnosed with migraine without aura that is not uncommon in clinical settings. In addition, to avoid making the cells numbers fewer we decided to include all cases of migraine with and without aura and probable migraine as migraine. The sensitivity was 0.87 and specificity was 0.58 (Table 2). In addition, the misclassification rate was 0.15, positive predictive value was 0.97, and negative predictive value was 0.26.

**Table 2:** SMI vs. headache specialist's clinical diagnoses "gold standard"

	Clinical Diagnoses		Total
**SMI Diagnoses**	**Migraine**	**No Migraine**	

Migraine	138 (a)	5 (b)	143

No Migraine	20 (c)	7 (d)	27

Total	158	12	170

Sensitivity: a/(a+c) = 138/138+20 = 138/158 = 0.87

Specificity: d/(b+d) = 7/5+7 = 7/12 = 0.58

Misclassification rate: b+c/(a+b+c+d) = 5+20/(138+5+20+7) = 25/170 = 0.15

Positive predictive value: a/(a+b) = 138/(138+5) = 138/143 = 0.97

Negative predictive value: d/(c+d) = 7/(20+7) = 7/27 = 0.26

With respect to comparing the face to face interview with an interviewer administered SMI telephone interview, from the 50 subjects selected; nine subjects were not contactable (disconnected telephone numbers or no longer residing at this address), one subject had died since participation in the original study and three subjects did not consent for future contact therefore were not contacted. Once 20 completed telephone interviews had been obtained no further subjects were contacted. Hence the final 17 subjects selected were not contacted and their data were excluded from the analysis. The spearman's rho correlation coefficient comparing the face-to-face and telephone interview methods for completing the SMI was 0.84, (p < 0.0001). Kappa statistic was 0.82 (p < 0.0001).

## Discussion

The migraine diagnosis generated by the structured migraine interview was positively associated with self reported migraine and receiving treatment for migraine in a clinical sample of headache sufferers attending a specialized clinic. It has been suggested that a disorder associated with impairment or requiring treatment is usually associated with good reliability of recall [30]. This may also reflect that the patients attending a specialized clinic for their headache represent the extreme end of severity and the associated impairment that they do require such specialist intervention. We can argue that these patients are highly selected with increased awareness about their headaches compared to general population samples. In addition, the retrospective collection of headache data has the disadvantage of recall bias that may lead to remembering the most severe episodes of headaches thus providing one extreme of the headache spectrum. Nonetheless the SMI was able to detect significant number of migraine sufferers in this sample. However, it cannot be concluded on the basis of the current study that self reported migraine or the use of treatment are accurate or reliable methods of diagnosing migraine.

The results of validity testing of the SMI show that compared to clinical diagnosis the SMI is highly sensitive (0.87) but less specific (0.58) in identifying migraine cases. This could be because almost all subjects referred to the migraine clinic (by definition) suffered from headaches so the validity sample was unbalanced by lack of subjects without headache. In addition, traditionally clinical diagnosis is known to be more liberal compared to structured criteria that were aimed at research case identification. In clinical practice some patients do not necessarily "fit in" to any classification system and the majority may have a combination of symptoms that need management regardless of the exact diagnosis especially for disorders such as migraine that are recurrent and disabling in nature. The difference between clinical and research diagnoses is therefore expected and in keeping with conventional practice. The questionnaire method, although restrictive, it is suitable to identify true cases of migraine that is paramount to research methods. Indeed, we have used the SMI in two large studies of depression case-control [23] where migraine prevalence in depressed subjects (n = 1259) was found significantly higher than psychiatrically healthy controls (n = 851) and a depression sibpair study (from 495 families, total subjects = 1052) where siblings concordant for depression showed positive correlations for migraine [31].

Although the numbers in the validity study are modest, we believe that the results are able to demonstrate specificity and sensitivity, and are in keeping with larger sample sizes [32]. A previous study employing similar methodology with a modest sample size of 61 headache sufferers concluded that a combination of using a diary and a clinical interviewing was more effective than either method alone [33]. In addition to the 170 individuals whose diagnoses were confirmed by the headache specialist in this study, 646 subjects who were attending the headache clinic and received formal diagnoses for their headaches were tested using the SMI. Even though the reported diagnoses were by self-report, the diagnoses obtained by the SMI were significantly correlated with self-reported migraine (Spearman's rho = 0.16, p = 0.001). In a study of self recognition of migraine, Lipton et al [34] noted that individuals who reported that they had migraine were 3 times more likely to meet the ICHD criteria for migraine.

A self administered questionnaire was tested in 713 individuals and compared to clinical interviews. Ninety three subjects were identified as having migraine in clinical interviews and 94 subjects classified as having migraine using the questionnaire method (a self administered questionnaire based on the ICHD 1988 criteria) [32]. The sensitivity of a self administered questionnaire in Rasmussen and colleagues study was lower than the SMI (0.51 compared to 0.87) with high specificity 0.92. The study using McNemar test for symmetry in a 2 × 2 table similar to table 2 found that the two types of inconsistencies (false positive by the questionnaire "b" and false positive by the clinical interview "c" were equally common (χ^2 ^= 0.01, df = 1, p < 0.9). The authors concluded that the use of self administered questionnaire was less sensitive but highly specific, therefore the use of self administered questionnaire in the clinical settings will lead to under-reporting of true cases of migraine [32]. Other studies have also shown significant sensitivity and specificity of self administered questionnaire based on the ICHD criteria for the diagnosis of migraine compared to clinical interview. In a German study of self administered headache questionnaire based on ICHD in a clinical setting validated against clinical interview, the sensitivity was 0.73 and specificity 0.96 [35]. In a Danish study of self administered headache questionnaire based on ICHD, which included 56 questions about headache and accompanying symptoms in a clinical sample, the sensitivity was 0.63 and specificity 0.92 for the over all diagnosis of migraine with and without aura [36]. Better validity parameters were achieved by looking at each type of migraine separately [36]. Other studies showed higher sensitivity (0.89) and lower specificity (0.79) of nurse administered (with no experience in headache disorders) migraine questionnaire based on the ICHD criteria and designed for use in clinical practice compared to a clinical interview by a headache specialist [37] reflecting the over inclusive nature of clinical diagnosis versus the narrow more stricter classification based diagnosis. A shorter self administered screening questionnaire based on 3 questions had also shown good sensitivity (0.81) and moderate specificity (0.75) but was unable to distinguish different types of migraine [27]. Our study demonstrated that the use of a structured interview (self rated or interviewer administered) based on the ICHD criteria is a useful and valid tool to use in research for the identification of migraine in large samples where clinical interviews are less practical. However, a questionnaire method can not replace clinical interviews when possible.

In this study, we have found that face-to-face interviews and telephone interviews showed significant correlations. However, the duration between the two interviews is relatively long (mean 2.5 years) to draw significant conclusions as during this period subjects may developed new onset of migraine or have recovered and forgot to report previous history of migraine. In addition the sample size in this part of the study is modest (n = 20) to produce firm conclusions. It was however exploratory and further work is needed to validate the SMI as a tool for telephone interviewing. It can also be argued that the repeatability of the original findings of migraine were probably accounted for by learned answers in that the subjects knew the questions and gave similar answers to the original questions. This scenario is unlikely given that the agreement was not 100% and the subjects were re-interviewed at various times. Studies of the validity of telephone interviews in comparison to face to face interviews for psychiatric disorders [38,39] and other medical conditions using different scales [40] have shown substantial agreement suggesting telephone interviews to be valuable tools in conducting research.

Other methods of migraine diagnosis were also tested such as the use of a headache diary [28]. The Copenhagen group employed similar study design to the SMI based on the IHCD 1988 criteria. The study included 61 participants from a specialized headache centre. The sensitivity of the headache diary for migraine with out aura was excellent at 0.94; the specificity however was modest at 0.50. For migraine with aura the sensitivity and specificity were 0.73 and 0.72 respectively. The authors concluded that the use of the diary should be supplemented by a clinical interview to improve accuracy [28]. The SMI had a sensitivity of 0.87 and specificity of 0.58 indicating a useful instrument in identifying migraine in settings where headache expertise is not available.

## Conclusions

The use of a questionnaire based on the ICHD criteria for migraine is shown to be a valid and simple method to use in diagnosing migraine in research settings and with caution in clinical settings due to limited specificity.

## Competing interests

The authors declare no competing interests. AF has received honoraria for presentations and chairing meetings from Eli Lilly, GSK and Wyeth and has acted as a consultant for GSK. PM has received honoraria from Eli Lilly and GSK and has acted as a consultant in the recent past for GSK and Astra Zeneca.

## Authors' contributions

ZS conceived the SMI idea and design, collected and analyzed data and wrote 1^st ^draft. EAM provided the clinical data, performed the diagnoses for the validity study and contributed to the writing of the manuscript. AD provided input into the SMI design and contributed to the manuscript writing. PM and AF supervised the overall study conduction, advised on the methods, statistical analysis, contributed to the manuscript and wrote the final draft. All authors read and approved the final manuscript.

## Pre-publication history

The pre-publication history for this paper can be accessed here:

http://www.biomedcentral.com/1471-2377/10/7/prepub

## Supplementary Material

Additional file 1 **Structured Migraine Interview**. The file contains the structured migraine interview ten questions. Click here for file
