# Supplementary material for: Diagnosing migraine in research and clinical settings: The validation of the Structured Migraine Interview (SMI)
Source: BMC Neurol. 2010 Jan 14;10:7. doi: 10.1186/1471-2377-10-7 (PMC2824671; doi:10.1186/1471-2377-10-7)
Supplement: Additional file 1 — Structured Migraine Interview. The file contains the structured migraine interview ten questions. [file 1471-2377-10-7-S1.DOC]

Date ___________ Name _______________ Date of Birth_________________ Gender: **** Male **** Female

1. Have you ever had recurrent headaches? **** No **** Yes

If yes, how old were you when these headaches started? ____ years

2. Have you ever had moderate to severe headache accompanied by nausea and/or vomiting? **** No never **** Yes 1-4 times **** Yes 5-9 times **** Yes 10+ times

3. Have you ever had moderate to severe headache accompanied by hypersensitivity to sound or light? **** No never **** Yes 1-4 times **** Yes 5-9 times **** Yes 10+ times

4. Have you ever had visual disturbances e.g. (Flashing lights, zigzag lines, blurred vision) Lasting 5-60 minutes followed by headache?

**** No never **** Yes once **** Yes 2+ times

**If answers to all questions 1-4 are “No” then finish here**

5. Typically what kind of headache do you get?

**** Pulsating **** Band like **** One-sided **** Other (specify):

6. Typically are your headaches

**** Mild (able to continue with daily activities)

**** Moderate (some limitations in daily activities)

**** Severe (unable to function)

7. What would make them worse? (can tick more than one)

**** Walking (or similar physical activity)

**** Certain food

**** Others (specify):

8. What do you do to relieve the headaches?

**** Rest **** Sleep **** Being in a dark room

**** Taking migraine tablets **** Taking pain-killers

**** Other (specify)

9. Typically how long do your headaches last?

**** 0-1 hour **** 2-3 hours **** 4-72 hours **** >72 hours

10. Have you been given a medical explanation and/or a diagnosis (including effects of medications you took) for your recurrent headaches? **** Yes **** No

If yes please state what was this explanation or diagnosis:
